# Supplementary figures and images for: A splice donor variant in CCDC189 is associated with asthenospermia in Nordic Red dairy cattle
Source: BMC Genomics. 2019 Apr 11;20:286. doi: 10.1186/s12864-019-5628-y (PMC6460654; doi:10.1186/s12864-019-5628-y)

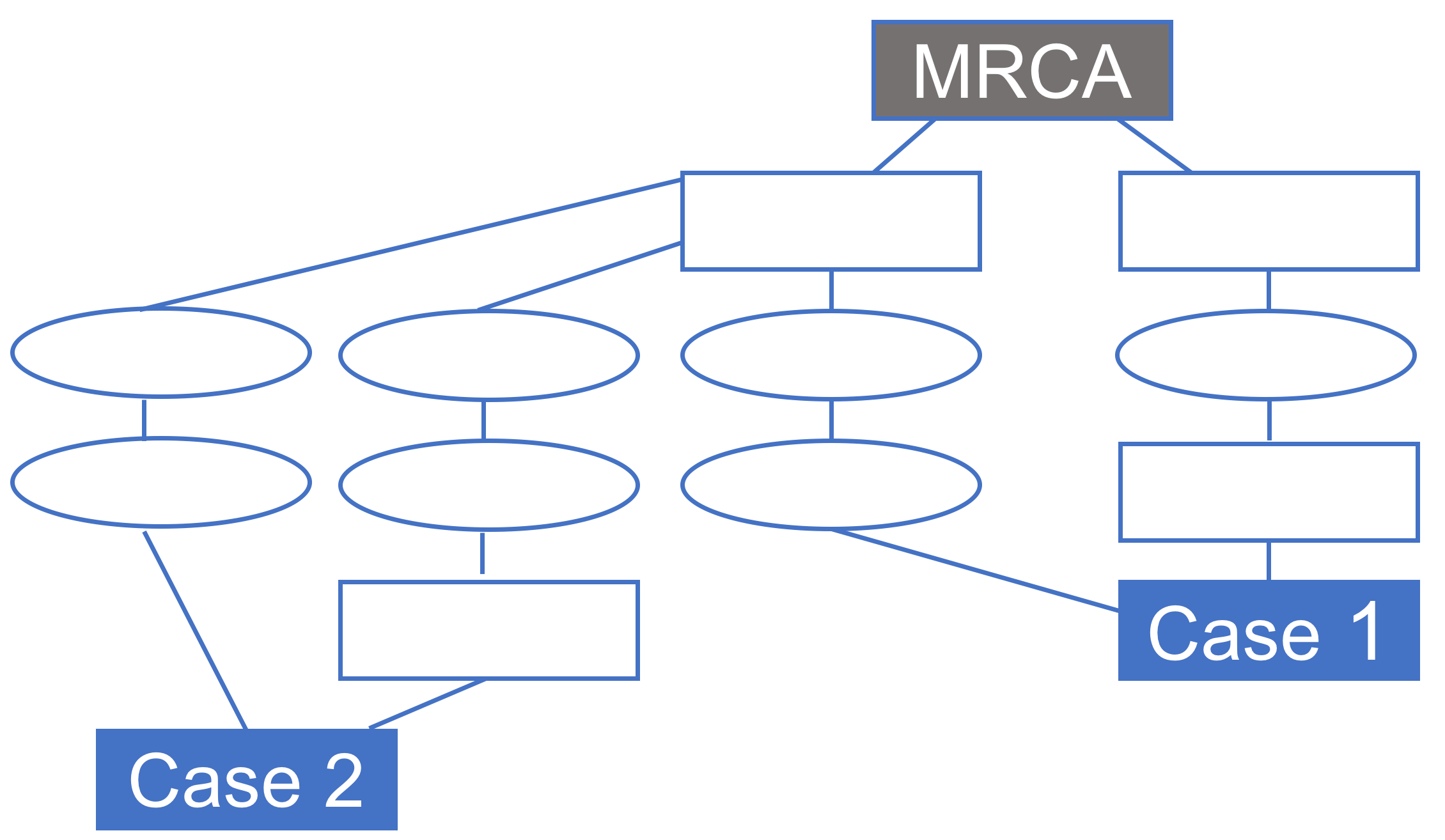

Supplement: Supplementary file 3 — Pedigree of two asthenospermic bulls. Pedigree of two affected bulls. Ovals and boxes represent female and male ancestors, respectively. The grey box highlights the most recent common ancestor (MRCA) that was present on both the maternal and paternal ancestry of both asthenospermic bulls. (PNG 199 kb) [file 12864_2019_5628_MOESM3_ESM.png]

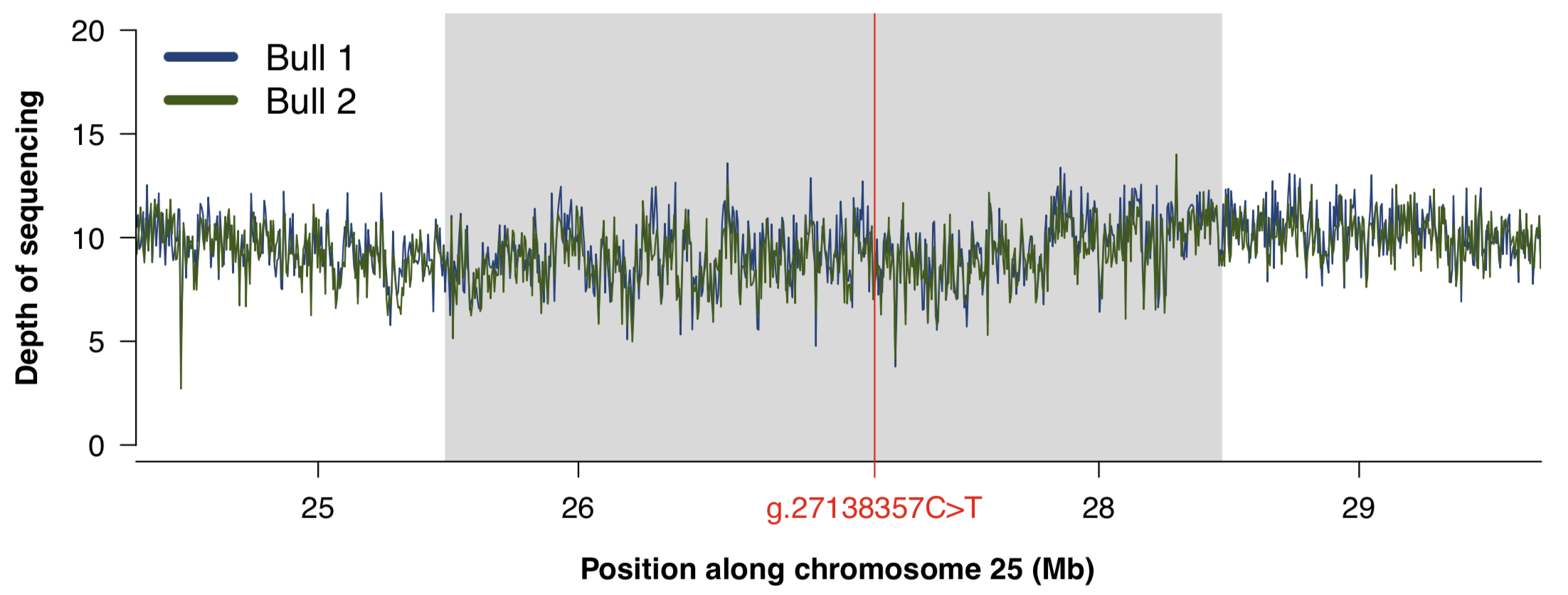

Supplement: Supplementary file 7 — Sequencing depth along chromosome 25 in two asthenospermic bulls. The depth of sequencing coverage in two asthenospermic bulls was calculated at bovine chromosome 25 in sliding windows of 5000 base pairs using the aligned reads. Grey background indicates the segment of extended homozygosity. The red line represents the position of the candidate causal variant- (PNG 604 kb) [file 12864_2019_5628_MOESM7_ESM.png]

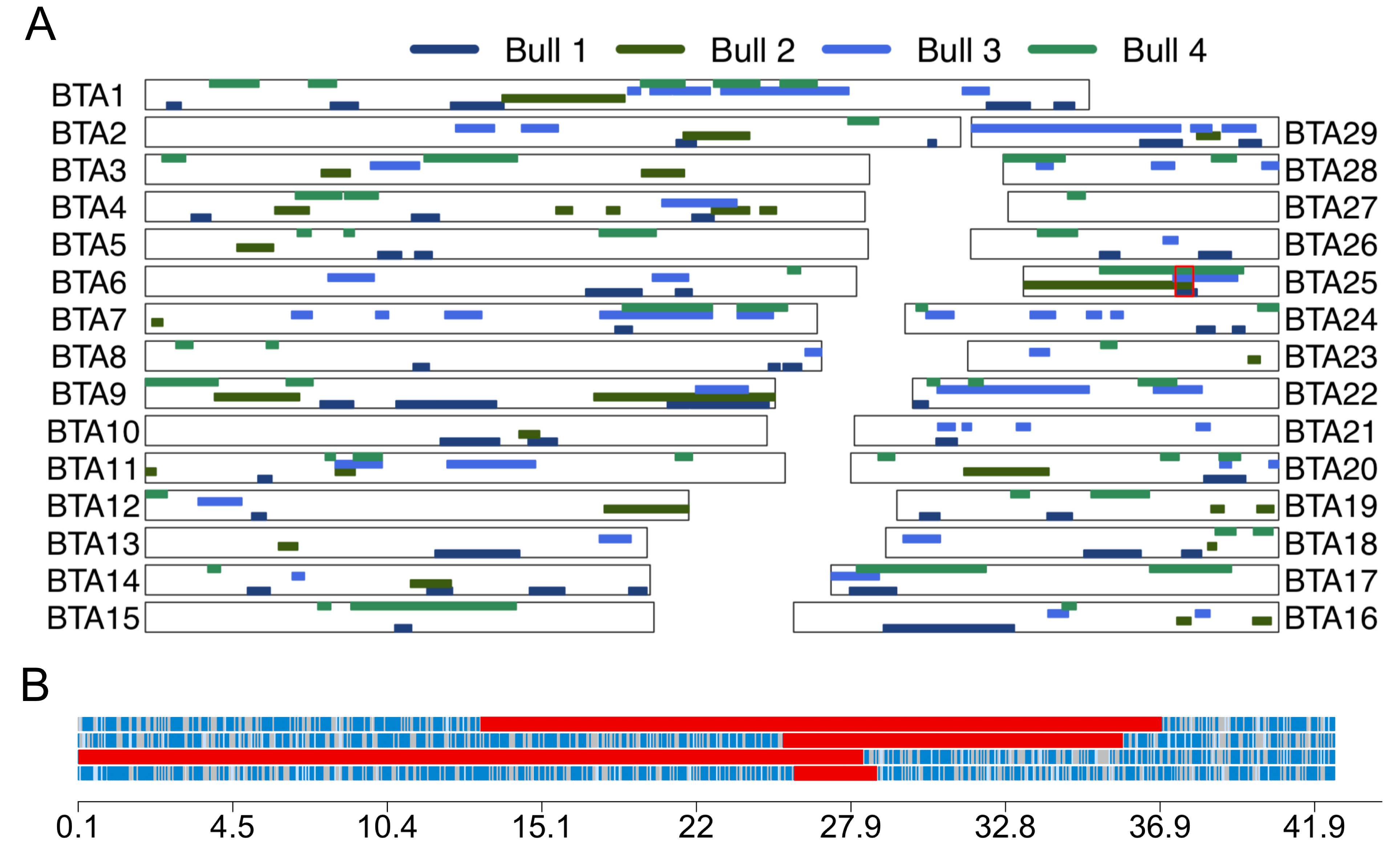

Supplement: Supplementary file 9 — Homozygosity mapping in four asthenospermic bulls. Homozygosity mapping in four asthenospermic bulls (A). Bulls 1 and 2 were already reported in the main part of the manuscript. Bulls 3 and 4 were additionally reported to us because they also produced immotile sperm. However, tissue or semen was not available for bulls 3 and 4. Blue and green color represent runs of homozygosity (ROH) that have been detected in four asthenospermic bulls. The red frame highlights the 2.98-Mb segment located on chromosome 25 that was never found in the homozygous state in normospermic bulls and that carries the splice donor variant in CCDC189. Autozygosity mapping on chromosome 25 (B). Blue and pale blue represent homozygous genotypes (AA and BB), heterozygous genotypes (AB) are displayed in light grey. White color indicates missing genotypes. The red bar indicates a common 2.98-Mb segment that was identical by descent in all four bulls. (PNG 1368 kb) [file 12864_2019_5628_MOESM9_ESM.png]
